# Parental Burnout and Early-Childhood Behavioral Problems: Longitudinal Associations Beyond Maternal Depression

**DOI:** 10.3390/children13020176

**Published:** 2026-01-27

**Authors:** Anna Suarez, Vera Yakupova

**Affiliations:** 1 Faculty of Psychology, Lomonosov Moscow State University, 125009 Moscow, Russia; vera.yakupova@msupress.com; 2 Federal Science Center of Psychological and Interdisciplinary Research, 125009 Moscow, Russia

**Keywords:** parental burnout, postpartum depression, maternal depression, child development, behavioral problems, internalizing problems, externalizing problems

## Abstract

**Highlights:**

**What are the main findings?**

- This was a 3-year follow-up study investigating the distinct adverse effect of parental burnout on child development while controlling for maternal depression.
- Parental burnout was significantly associated with child internalizing and externalizing problems, independently of postpartum or present maternal depression at the dimensional level, even though associations with borderline/clinically significant outcomes were attenuated once overlapping maternal mental health factors were considered.

**What are the implication of the main findings?**

- Parental burnout presents a unique risk factor for child adverse outcomes in toddlerhood, distinct from maternal depression, which requires further research.
- It is crucial to recognize and support families where parents are experiencing significant burnout and caregiving-related exhaustion, particularly in contexts of limited social and institutional resources, regardless of the presence of a formal mental health diagnosis.

**Abstract:**

Background: Parenting is increasingly recognized as a highly demanding and stressful role that, in the absence of sufficient resources, may lead to parental burnout (PB). This risk may be particularly pronounced in the Russian context, where limited access to childcare for children under three and reduced extended family support coincide with strong social expectations of intensive parenting. Although PB and maternal depression frequently co-occur, it remains unclear whether PB exerts a unique influence on child development, especially during toddlerhood. The present study examined the association between PB and behavioral problems in children aged 1.5 to 4 years while controlling for maternal depression assessed both during the first year postpartum and concurrently with PB. Methods: Using a longitudinal design, maternal mental health was assessed within the first 12 months postpartum (Stage 1) and again at follow-up (Stage 2), on average 2.24 years later, in 419 Russian mother–child dyads. Mothers completed measures of postpartum depression (PPD) (Edinburgh Postnatal Depression Scale), current depressive symptoms (Beck Depression Inventory-II), and PB (Parental Burnout Inventory). Child emotional and behavioral problems were assessed at Stage 2 using the Russian version of the Child Behavior Checklist (CBCL/1½–5). Results: Mothers of children with borderline/clinically significant internalizing, externalizing, and total problems had significantly higher PB, PPD, and present maternal depressive symptoms, although the effect sizes were small. PB was strongly associated with all domains of child behavioral problems, also after correction for both postpartum and present depressive symptoms, as well as for other important covariates. Higher maternal PB symptoms further increased the odds of children having borderline/clinically significant internalizing and externalizing problems, although those effects were not independent of maternal depression. In turn, neither postpartum nor present maternal depressive symptoms were associated with any of the child behavioral problems domains. Conclusions: PB represents a distinct and clinically relevant risk factor for emotional and behavioral problems in toddlers, beyond the effects of maternal postpartum or present depression, in a context characterized by high caregiving demands and limited institutional support. These findings highlight an urgent need for programs aimed at identifying and supporting families in which parents experience high levels of exhaustion, regardless of whether they meet the criteria for other diagnosable mental health disorders. Addressing PB during toddlerhood may be critical for protecting both parental well-being and early child development.

## 1. Introduction

### 1.1. Background

Parenting is widely regarded as one of the most valuable human experiences, yet it is increasingly acknowledged as a demanding role accompanied by persistent stress. This phenomenon is often observed in Western cultures, where individualistic values foster heightened expectations of parents [1]. When these expectations exceed the resources available to parents, they may develop symptoms of parental burnout (PB). PB is characterized by physical and mental exhaustion, including somatic complaints and sleep disturbances, emotional distancing from children, and feelings of inadequacy in fulfilling parental responsibilities [2]. Preliminary investigations have identified various consequences of PB, such as escape ideations, suicidal thoughts, sleep disorders, addictions, marital conflicts, and instances of child abuse or neglect [3]. Factors contributing to PB include unrealistic expectations of parenting, insufficient support from partners or caregivers, and particular parental or child personality traits, such as high neuroticism [3,4].

The distinction between burnout and depression remains debated due to overlapping symptoms and diagnostic challenges, including sleep problems, substance use, somatic health issues, and relational conflicts [5,6,7]. However, several studies indicate that these constructs are related yet distinct. Specifically, low to moderate correlations have been reported between PB, work-related burnout, parental stress, and depressive mood [4,8]. Emerging evidence further suggests that the symptoms and consequences of PB and parental depression differ substantially [9,10]. Unlike depressive symptoms, PB is strongly associated with low parental satisfaction, neglect, and violence toward children [3] and appears to primarily affect family dynamics, whereas depression has broader impacts across multiple life domains [9]. These findings underscore the need for further research to clarify PB’s unique implications for parental and child well-being.

Most PB studies have focused on mothers, who are often regarded as primary caregivers, particularly during the postpartum period [11,12]. Séjourné et al. identified a significant association between maternal burnout and a history of postpartum depression (PPD) [13]. Their findings also linked PB to anxiety, perceptions of a child as difficult, dissatisfaction with work–life balance, and parental stress. These results are consistent with cross-sectional studies conducted on 560 mothers in China and 1300 French-speaking mothers, both of which reported a positive association between PPD and PB [12,14]. However, longitudinal research examining depressive symptoms during both the postpartum period and later stages of parenthood in relation to PB remains limited.

PB affects not only parental well-being but also child development. Two studies from Russia found a positive association between PB and preschoolers’ emotional and cognitive development, reporting improved executive functions and emotional comprehension skills in children of mothers with higher PB symptoms, independent of maternal depression [15,16]. Conversely, other studies have linked higher PB symptoms to increased internalizing and externalizing problems in Chilean preschoolers [17] and Chinese adolescents [11]. These discrepancies may reflect cultural differences in norms of intensive, child-centered parenting, as well as developmental stages, with PB potentially signaling overinvestment among Russian preschool mothers but reduced emotional availability and harsher parenting in other sociocultural contexts and at later ages. Therefore, further studies are warranted to explore the relationship between PB and child mental health problems during different stages of development and cultural contexts.

### 1.2. Russia-Specific Context

According to international consortium data, Russia is considered a country with high risks for PB exposure [1]. Consistent with the broader literature that has largely focused on parents of children aged four and older [17,18,19], Russian studies to date have been limited to parents of preschoolers [15,16] and primary school children [20]. Consequently, little is known about PB symptoms among Russian parents of toddlers.

The present study addresses this gap by focusing on the well-being of parents of toddlers, thereby extending knowledge on PB and its relation to child development in the earliest years. Russian parents may be particularly vulnerable to PB during toddlerhood, as this stage combines structural challenges—such as limited extended family support and the absence of free childcare for children under age three [21]—with increasing social pressures toward intensive parenting and high standards of “good parenting” [20,22].

### 1.3. Aims of the Study

Building on evidence that PB represents a parenting-specific form of distress with potential implications for early child development [23], the present study examines the association between maternal PB and socioemotional and behavioral problems in children aged 1.5 to 4 years, a sensitive developmental period. Using a longitudinal design, we aimed to assess whether PB is associated with child internalizing, externalizing, and total behavioral problems independently of maternal depressive symptoms assessed both during the first year postpartum and concurrently at follow-up. We hypothesized that higher levels of PB would be associated with greater child behavioral problems across all domains, beyond the effects of postpartum and current maternal depression.

## 2. Materials and Methods

### 2.1. Procedure and Participants

The present study employed a longitudinal design. At Stage 1, data collection occurred in two waves: February–March 2020 and February–March 2021. Participants were recruited through multiple channels, including childbirth education courses, maternity care providers (such as physicians and midwives), online forums and social media groups for expectant and new parents, and perinatal health professionals. Altogether, 2256 Russian women completed the online survey across these two time points.

Eligibility criteria required that participants be at least 18 years old, literate in Russian, have given birth to a live infant within the past 12 months, and that the delivery occurred in Russia. The data was collected from different regions of Russia (Central region, Ural, Tatarstan, North and South regions), predominantly from big cities, i.e., Moscow, St. Petersburg, Ekaterinburg, Kazan, Novosibirsk, Ufa, Krasnodar.

Between August 2022 and July 2023, those who had previously participated and expressed interest in being contacted for continuous participation in the study were contacted via email and invited to join the follow-up phase (Stage 2). Respondents were mailed printed questionnaires. Stage 2 focused on assessing maternal mental health and child developmental outcomes 1.5–4 years after childbirth. Of the original cohort, 589 women returned the completed follow-up forms. Following data verification procedures—including matching Stage 1 and Stage 2 identifiers, removing duplicates, and excluding incomplete responses—419 mother–child dyads with complete datasets were retained for analysis. The retention rate was 18.5%. There were no statistically significant differences between women who chose to participate at Stage 2 of the study and those who did not in terms of EPDS scores, maternal age, marital status, mode of birth, and child gestational age at birth (*p*-values for all > 0.41). There were statistically significant differences in the levels of education (with slightly more women with higher education among the participants who answered at Stage 2 (90.8% vs. 95.5%, *p* = 0.010), child medical complications during pregnancy/childbirth (with mothers who participated at Stage 2 reporting their children having medical complications during pregnancy/childbirth more often (14.6% vs. 20.5%, *p* = 0.023), and parity (there were more first-time mothers among those who participated at Stage 2 (52.5% vs. 60.1%, *p* = 0.020).

The Ethical Committee of the Russian Psychological Society at Lomonosov Moscow State University approved the present study (No: 345/2019, 6 December 2021). All participants provided their informed consent using the online tool Testograph prior to filling in the survey. The study was conducted in accordance with the Declaration of Helsinki.

### 2.2. Measures

#### 2.2.1. The Demographic Questionnaire

The online survey included questions regarding the participants’ age at the time of testing, highest achieved level of education (primary/secondary or tertiary) and marital status (married/cohabiting with partner or single/divorced), gestational age at childbirth, the number of children in a family, mode of birth (vaginal (assisted vaginal)/cesarean (planned/emergency)), and perceived socioeconomic status in comparisons to other residents of their current region (low/middle/high). We also collected the data about the child’s age at Stage 2 and the child’s complications during pregnancy/childbirth (yes/no).

#### 2.2.2. Parental Burnout

We used the Russian version of the Parental Burnout Inventory (PBI) [24,25] to analyze the level of maternal PB. The PBI is a 23-item questionnaire assessing four core symptoms of PB: emotional exhaustion (9 items; e.g., I feel completely run down by my role as a parent), contrast with previous parental self (6 items; e.g., I tell myself I’m no longer the parent I used to be), loss of pleasure in one’s parental role (5 items; e.g., I do not enjoy being with my children), and emotional distancing from one’s children (3 items; e.g., I am no longer able to show my children that I love them) [24]. The participants were asked to assess each statement using a seven-point frequency scale ranging from 0 to 6 (never, a few times a year, once a month or less, a few times a month, once a week, a few times a week, every day). The PB score is computed by summing the item scores: higher scores reflect higher parental burnout levels. The cut-off point for PB is 74 [26]. The internal consistency of the Russian version of the scale was Cronbach’s alpha α = 0.97 [25], Cronbach’s alpha for this sample was α = 0.98.

#### 2.2.3. Edinburgh Postnatal Depression Scale

The Russian version (Cronbach’s α = 0.84) of the Edinburgh Postnatal Depression Scale (EPDS) was used to estimate PPD symptoms [27,28]. It is a 10-item questionnaire scale rated on a 4-point Likert scale, ranging from 0 to 3, which indicates how the mother has felt during the previous week. A score of 10 and higher is considered to indicate clinically significant symptoms of depression [27]. Cronbach’s alpha for the study sample was α = 0.88.

#### 2.2.4. Beck Depression Inventory

The Russian version of the Beck Depression Inventory (BDI-II) was used to assess the levels of parental depression [29,30]. The BDI-II is a 21-item, self-report questionnaire measuring symptoms of depression [29]. Items are scored on a scale from 0 to 3, ranging from 0: “I do not feel guilty,” 1: “I often feel guilty”, and 2: “I feel guilty most of the time,” to 3: “I feel guilty all the time”. The depression score is obtained by summing the 21 item scores, with a score of 20 and higher indicating moderate and severe symptoms of depression [29]. For the Russian version, Cronbach’s coefficient is α = 0.87 [30], with Cronbach’s coefficient α = 0.89 in this sample.

#### 2.2.5. Child Behavior Problems and Emotional Development

To study emotional and behavioral problems in children, the Russian version of the Child Behavior Checklist (CBCL/1½–5) was used [31,32]. It includes 99 statements describing various deviations in the behavior and emotional state of children. The child’s parent marks each statement as incorrect (0), sometimes true or partially true (1), or very true (2). The raw scores were translated into T-scores, with T ≥ 65 indicating a borderline level of severity of the problem and T ≥ 70 corresponding to a clinical level of behavioral problems [31]. The CBCL/1½–5 behavior checklist evaluates three domains of behavioral problems: internalizing (emotional reactivity, anxiety/depression, somatic complaints, withdrawn symptoms), externalizing (attention problems, aggressive behavior), and total problems. Furthermore, there are five DSM-oriented scales (depressive problems, anxiety problems, autism spectrum problems, attention deficit/hyperactivity problems, oppositional defiant problems) that reflect psychological and behavioral syndromes aligned with diagnostic categories from the Diagnostic and Statistical Manual of Mental Disorders (DSM-V) [31]. The internal validity of the three domain scales in the Russian version is Cronbach’s α = 0.91, α = 0.94, and α = 0.97 for internalizing, externalizing, and total problems, respectively [32]. In the present sample, Cronbach’s α = 0.82 for internalizing, α = 0.87 for externalizing, and α = 0.90 for total problems.

### 2.3. Covariates

Statistical models were adjusted for maternal age at testing, level of education, family status, socioeconomic status, the number of children in a family, mode of birth, gestational age at childbirth, child’s complications during pregnancy/childbirth, and child’s age at Stage 2 as covariates.

### 2.4. Statistical Analysis

For all the analyses, maternal depressive symptom scores (both postpartum and present) were square root-transformed, and PB and CBCL scores were log-transformed to attain normality; the symptom scores were further standardized to a mean of 0 and an SD of 1 to facilitate interpretation.

Spearman correlation analysis was used to examine the correlation between raw PB scores, postpartum and present depressive symptoms, and child behavioral problems and DSM-oriented scales. Although variables were transformed for parametric modeling, Spearman rank-order correlations were retained for bivariate analyses because the measures represent symptom severity scales and did not fully meet normality assumptions even after transformation. Spearman correlations were therefore used as a conservative, distribution-free descriptive approach, while transformed variables were applied in subsequent parametric analyses.

A one-way ANOVA was used to explore association of maternal PB, PPD, and present depressive symptoms with CBCL scores. After transformation, assumptions for parametric analyses were formally evaluated. Homogeneity of variance was assessed using Levene’s test and was supported for the transformed variables (*p* = 0.96). Normality was examined using skewness indices and visual inspection of residual distributions.

Multiple linear regression analysis examined the association of PB scores with continuous child behavioral problem scores (internalizing, externalizing, and total problems as well as DSM-oriented scales), while binary logistic regression tested the association between maternal PB and borderline/clinical levels of child internalizing, externalizing, and total problems (yes/no).

For both groups of parametric analyses four models were tested: Model 1 was adjusted for the covariates listed above. Model 2 was further adjusted for PPD symptoms, while Model 3 included the covariates from Model 1 and present maternal depressive symptoms.

All statistical analyses were conducted using SPSS software, version 27 (IBM Corp, NY, USA, 2020) [33].

## 3. Results

The demographic characteristics of the mothers and their children are presented in Table 1. The data indicate that the majority of mothers originate from middle-income families (58.9%), are married or have a partner (95.2%), have higher education (95.5%), and have one child (60.1%). Our sample further consisted of young children with a mean age of 2.24 years, most of whom had no reported complications during pregnancy or childbirth, although approximately one fifth experienced at least one complication (Table 1).

Table 1 shows that, among the participants, 10.3% of mothers reported clinically significant current depressive symptoms, while 35% showed clinically significant symptoms of PPD and 16.2% of mothers reported PBI scores above the cut-off point. PB scores were highly correlated with present depressive symptoms and moderately correlated with PPD (Spearman correlation coefficient = 0.65 and 0.36, respectively, *p* < 0.001 for both) (Supplemental Table S1).

Spearman correlation analysis further showed that all the CBCL scales correlated strongly with PB scores and with postpartum and present depressive symptoms (*p* < 0.01), with two exceptions: there was only a modest correlation between the DSM-oriented autism problems scale and present maternal depression (rho = 0.11, *p* = 0.03), while there was no significant correlation between the same CBCL scale and PPD symptoms (rho = 0.08, *p* = 0.095) (Supplemental Table S1).

**Figure 1:**
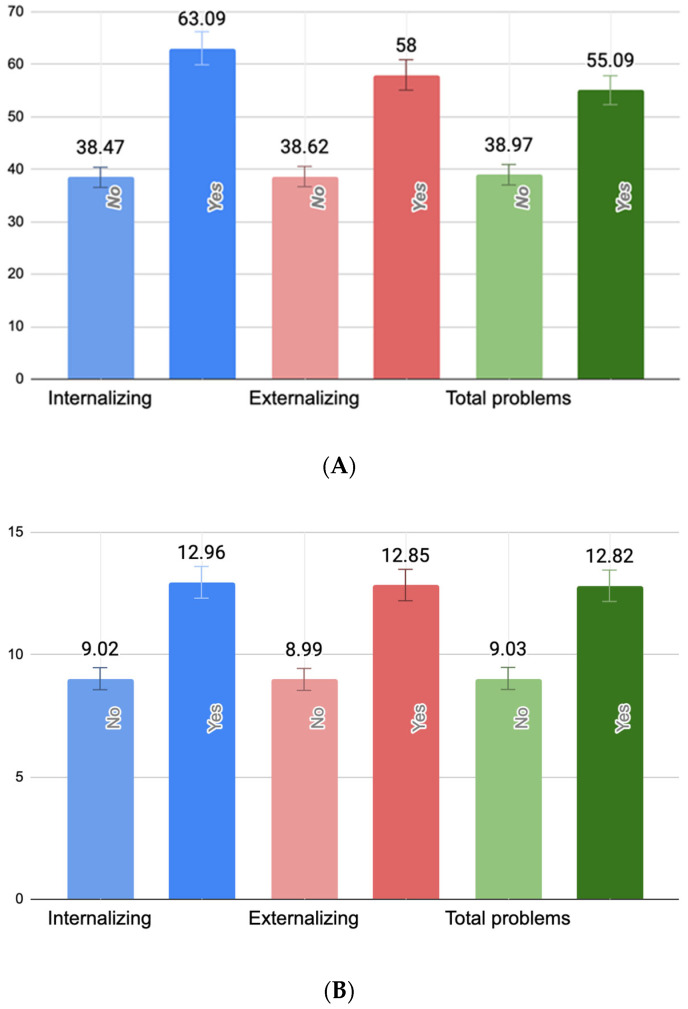

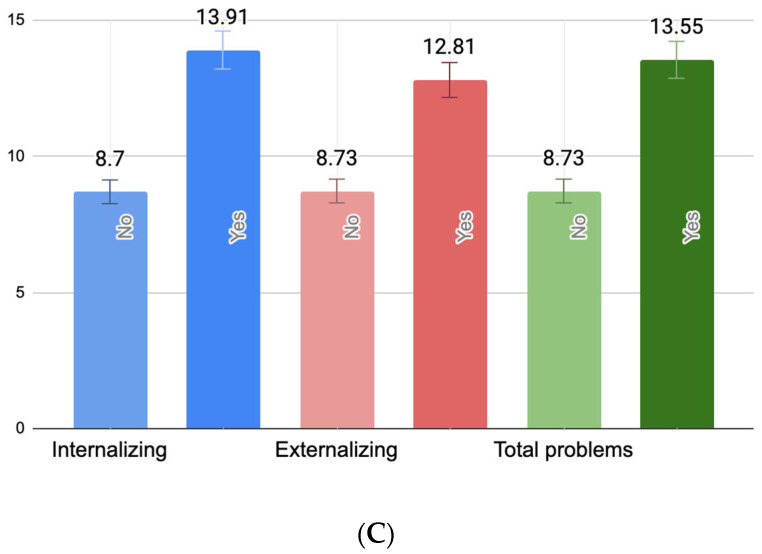
Mean levels of maternal parental burnout (**A**), postpartum depressive symptoms (**B**), and present maternal depressive symptoms (**C**) by child behavioral problem status. (**A**) Mean differences between parental burnout symptoms (raw PBI scores) in mothers of children with and without borderline/clinically significant behavioral problems. (**B**) Mean differences between postpartum depression symptoms (raw EPDS scores) in mothers of children with and without borderline/clinically significant behavioral problems. (**C**) Mean differences between present maternal depressive symptoms (raw BDI-II scores) in mothers of children with and without borderline/clinically significant behavioral problems.

One-way ANOVAs revealed statistically significant associations between PB, PPD, and current maternal depressive symptoms and all three domains of child behavioral problems. Specifically, PB scores were significantly higher among mothers of children with borderline/clinically significant internalizing (F(1418) = 5.03, *p* = 0.025), externalizing (F(1418) = 7.35, *p* < 0.01), but not total behavioral problems (F(1418) = 3.22, *p* = 0.074). Similarly, maternal PPD symptoms were significantly elevated in cases of child internalizing (F(1418) = 8.18, *p* < 0.01), externalizing (F(1418) = 9.38, *p* < 0.01), and total problems (F(1418) = 7.76, *p* < 0.01). Current maternal depressive symptoms were also significantly higher among mothers of children with borderline/clinically significant internalizing (F(1418) = 9.91, *p* < 0.01), externalizing (F(1418) = 8.12, *p* < 0.01), and total behavioral problems (F(1418) = 8.74, *p* < 0.01). Figure 1 illustrates these group differences. Importantly, the figure reflects untransformed scores of PBI, EPDS, and BDI-II scores for ease of clinical interpretation.

Table 2 shows that PB is strongly associated with all the scales of child behavioral problems. Importantly, the association remains significant in the models additionally adjusted for both postpartum and present maternal depressive symptoms. In turn, neither PPD nor present maternal depression were significantly associated with any of the outcomes (Table 2).

Table 3 demonstrates statistically significant associations between PB and child borderline/clinically significant internalizing and externalizing behavioral problems. Specifically, higher levels of PB were associated with a 70% increase in the odds of internalizing problems (OR = 1.70, 95% CI [1.03, 2.82]) and a 74% increase in the odds of externalizing problems (OR = 1.74, 95% CI [1.05, 2.87]). These effect sizes indicate small-to-moderate associations. However, these associations were attenuated after adjustment for postpartum or current maternal depressive symptoms. In turn, neither postpartum nor present maternal depressive symptoms were associated with any of the child behavioral problems domains (Table 3).

## 4. Discussion

The present study examined the associations between maternal psychological distress, namely, PB, PPD, and present depression, and child behavioral and emotional problems, as measured by the CBCL/1½–5, in children aged 1.5 to 3.5 years. The longitudinal design allowed us to control for PPD measured during the first year postpartum while simultaneously assessing PB and present depressive symptoms at 2.24 years later. This constitutes an important methodological advancement, as previous research linking PB to child internalizing and externalizing problems rarely adjusted for either postpartum or concurrent maternal depression [17,34]. Overall, our findings underscore significant associations between maternal mental health and child outcomes and provide novel evidence for the distinct contribution of PB to early childhood behavioral problems.

In our sample, PB was prevalent in 16.2% of mothers, substantially higher than the 2.3% previously reported among Russian parents of preschoolers [16]. This suggests that toddlerhood, like adolescence, may represent a particularly vulnerable developmental period for parental exhaustion in Russia.

This heightened vulnerability likely stems from structural and cultural pressures that are especially pronounced during the toddler years. Unlike parents of preschoolers, parents of toddlers in Russia typically lack access to free public childcare for children under three, and private options are often financially inaccessible. At the same time, extended family support has become less accessible due to urbanization and geographic mobility, reducing opportunities for shared caregiving. These constraints coincide with intensified societal expectations of “good parenting”, which emphasize constant engagement, developmental stimulation, and high emotional availability [34]. Together, these factors place substantial and sustained demands on parents of toddlers, potentially explaining why burnout appears more common in this group than among parents of older children [35].

Despite a comparable or even higher prevalence than maternal depression, however, PB remains unrecognized in Russian clinical guidelines. The absence of systematic screening, professional training, or specialized support services likely contributes to underdiagnosis and leaves many mothers without necessary psychological assistance.

This lack of structured support systems may amplify the impact of PB on both maternal well-being and child outcomes, highlighting the pressing need for policy and clinical frameworks targeting parental exhaustion. In line with our hypothesis, the regression models revealed significant associations between PB and all domains of child behavioral problems, even after controlling for both postpartum and present maternal depressive symptoms. This pattern reinforces prior evidence that PB uniquely contributes to child maladjustment [17,36]. Interestingly, findings from Russian samples of preschool-aged children have shown a different pattern, with higher maternal PB being associated with better emotional and cognitive outcomes in children, independently of maternal depression [15,16]. The heterogeneity of findings across studies examining PB and child outcomes may, therefore, reflect important moderating factors. In particular, child developmental stages may shape how parental exhaustion affects children: during toddlerhood, children are highly dependent on primary caregivers and possess limited self-regulatory capacities, which may render them more vulnerable to reduced parental emotional availability. In contrast, among preschool-aged children, greater autonomy and engagement with broader social environments may alter the ways in which parental exhaustion is expressed and experienced, giving rise to different interactional pathways. In the Russian context, higher parental burnout in mothers of preschoolers may reflect sustained overinvestment in children’s development rather than reduced caregiving quality, consistent with strong cultural norms encouraging intensive, child-centered parenting [15,16]. It is also plausible that specific parenting behaviors, such as positive involvement or psychologically intrusive control, mediate or moderate these associations; however, such mechanisms were not assessed in the present study and warrant dedicated investigation in future work.

When examining borderline or clinically significant child behavioral problems, we found that mothers of children with difficulties above the cut-off reported higher levels of PB, PPD, and present depressive symptoms across the board. As a result, the associations between PB and clinically significant/borderline internalizing and externalizing problems in binary regression analyses were attenuated once overlapping maternal mental health factors were considered. This might suggest that in cases of more severe child difficulties, multiple maternal mental health challenges co-occur, making it harder to isolate the unique contribution of PB [37]. Furthermore, it is possible that PB may be particularly relevant for explaining subthreshold and mild-to-moderate child behavioral problems, whereas more severe psychopathology likely reflects a broader constellation of maternal mental health difficulties. In other words, PB may capture parenting-specific strain that is closely tied to everyday child behavior, while depression and other comorbid conditions may become more salient as predictors when child behavior reaches clinical levels of impairment. PB may influence child outcomes primarily through changes in parenting behavior, such as reduced emotional availability, harsher or more inconsistent discipline, and increased psychological intrusion or control, which have been linked to higher internalizing and externalizing symptoms in children. While emerging longitudinal work suggests that such parenting strategies can partly mediate the association between PB and child adjustment, these mechanisms remain preliminarily tested only in parent–adolescent dyads [38].

Crucially, these relationships are likely bi-directional [39,40]. Children exhibiting emotional or behavioral difficulties may place additional demands on parents, increasing parental exhaustion and PB. Conversely, parents experiencing burnout may be less responsive or emotionally available, which can further exacerbate children’s vulnerability to internalizing and externalizing symptoms [17]. While these effects are particularly pronounced for parents of children with more severe problems, evidence indicates that all parents benefit from support, regardless of the severity of child difficulties [41].

Contrarily, neither postpartum nor current maternal depressive symptoms were significantly associated with child behavioral outcomes once PB was accounted for. While this may appear counter to prior research [42,43,44], including findings from the same longitudinal cohort in which children of mothers with persistently high depressive symptoms exhibited pronounced behavioral problems [45], several explanations are possible. Current depressive symptoms were highly correlated with PB (rho = 0.65), indicating substantial overlap. It is therefore plausible that maternal depression influences child behavior indirectly via PB, with regression models isolating the variance specifically attributable to parental exhaustion, the aspect most proximally linked to child outcomes. PB is uniquely characterized by fatigue, emotional depletion, and reduced emotional availability in the parent–child relationship. These symptoms can lead to less responsive and more punitive parenting, which, in turn, may increase child internalizing and externalizing problems [34,46,47]. Prior research further demonstrates that PB predicts child maltreatment, including verbal aggression and neglect [3,36], both strong risk factors for child behavioral difficulties [48,49,50]. Unlike depressive symptoms, which may arise from diverse life stressors, PB is specific to parenting demands and captures the unique burden of parental responsibilities. Importantly, research on PB remains limited; while maternal depression and child development have been extensively studied for decades, few studies have explicitly examined PB or accounted for its influence independently. Consequently, the current findings highlight PB as a key proximal mechanism linking maternal mental health and child outcomes and the need for further studies in this field.

Thus, it is crucial to recognize PB as a distinct and clinically significant risk factor, implement systematic screening in pediatric and maternal healthcare settings, and develop targeted interventions aimed at reducing parental exhaustion to safeguard both maternal well-being and child developmental outcomes.

### 4.1. Strengths and Limitations

The strengths of our study include the longitudinal study design, which allows us to uniquely estimate the association of maternal PB and child behavioral problems while controlling for PPD symptoms measured within 12 months after childbirth, thus allowing us to evaluate the effects of PB, PPD, and present maternal depression independent of each other. The use of validated questionnaires and controlling for a number of significant covariates (such as child’s gestational age at birth, child health complications during pregnancy/postpartum, maternal education, and socioeconomic status) can also be considered as strengths. Particularly, the use of the CBCL/1½–5, a well-validated and internationally standardized instrument, allowed for comprehensive assessment of both internalizing and externalizing behavioral problems in early childhood and facilitated comparison of our findings with existing international research [31].

Nonetheless, certain limitations must be acknowledged.

First, child behavioral data were obtained exclusively through maternal reports, which introduces the possibility of reporter bias, particularly in studies involving parental mental health. Although the CBCL/1½–5 is a reliable and widely used instrument designed to minimize subjective interpretation, maternal perceptions may still be influenced by emotional states such as burnout or depressive symptoms. This risk is inherent to research conducted in early childhood contexts where mothers are typically the primary, and often the sole, caregivers, which is the case in Russia, where alternative informants are rarely available for children under three years old. While our analyses did not identify significant associations between maternal depression and child behavioral problems after accounting for PB, reliance on a single informant nevertheless limits the interpretability of the directionality of the effects.

Second, we observed a high attrition rate: only 18.5% of participants from Stage 1 returned the completed surveys at Stage 2. Although substantial dropout is common in longitudinal research, in our study, it may have been exacerbated by the use of printed CBCL/1½–5 questionnaires mailed to parents. This shift from an online format was necessary because the researchers who validated the CBCL/1½–5 in Russia had obtained distribution rights only for the printed version. As a result, the change in administration mode may have contributed to lower response rates.

Furthermore, sample attrition may also have been influenced by the high level of stigma surrounding maternal mental health in the context of child development in Russia. Sociological research shows that Russian society is predominantly child-centered, with strong social expectations that parents, particularly mothers, prioritize their children’s needs above their own [51,52]. Consequently, when women were invited to participate in a study that included questions about both child behavioral difficulties and their own mental health, many may have been reluctant to disclose personal struggles. Future research would benefit from improved access to more vulnerable groups, alongside broader public awareness and psychoeducational efforts aimed at reducing the stigma around parental mental health.

Importantly, the follow-up sample did not differ in PPD scores from the baseline sample, allowing us to examine groups with comparable levels of vulnerability. Notably, a higher proportion of first-time mothers (52.5% vs. 60.1%) and mothers of children with medical complications during pregnancy/childbirth (14.6% vs. 20.5%) participated at Stage 2. This may reflect heightened concern about their children’s development, as well as possible increased caregiving challenges and associated fatigue.

Finally, our findings are limited by the absence of objective medical data on maternal mental health at both stages of the study, as well as obstetric and child medical information. Instead, the study relies solely on self-reported data—a common limitation in contexts such as Russia, where registry-based medical records are either unavailable or not publicly accessible [49].

### 4.2. Further Directions

Future research should aim to build on the present findings by addressing several methodological and conceptual limitations. First, expanding the sample size and enhancing representativeness is essential to improve external validity. Studies including fathers, families from diverse socioeconomic backgrounds, and participants from different cultural and regional contexts would allow for a more comprehensive understanding of PB and its role in child development.

Second, future studies would benefit from incorporating additional informants and methodologies to reduce the shared method bias inherent in self-report designs. Collecting data from fathers, other caregivers, teachers, or healthcare professionals, as well as using observational assessments of parent–child interactions or clinician-rated measures of child behavior, would strengthen the objectivity and robustness of findings. Although such approaches are currently challenging in early childhood contexts in Russia, their inclusion represents an important goal for future research efforts.

Third, to clarify the directionality and causal mechanisms linking PB and child behavioral outcomes, future longitudinal studies should include additional measurement waves and apply cross-lagged panel or other dynamic modeling approaches. This would allow for examination of reciprocal effects between parental exhaustion and child behavior over time.

Finally, future research should consider a broader range of contextual and individual confounders, such as parenting styles, marital satisfaction, social support, child temperament, and parental coping strategies. Examining protective factors alongside risk mechanisms may help identify pathways through which PB affects child development and inform the design of targeted preventive and intervention programs.

## 5. Conclusions

The present work contributes to understanding the unique role of PB in early childhood development, showing that PB is associated with all domains of child behavioral problems independently of postpartum or present maternal depression at the dimensional level, even though associations with borderline/clinical outcomes were attenuated once overlapping maternal mental health factors were considered. Unlike much previous research, our longitudinal design allowed us to account for both postpartum and concurrent depressive symptoms, providing clearer evidence that exhaustion from parental responsibilities alone can negatively affect children. These findings underscore the importance of recognizing and addressing PB, even in the absence of depression, and point to a high need for emotional and practical support for parents, particularly during the toddler years, when parenting demands are especially intense and designated help (e.g., accessible childcare, extended family support) is often lacking, as in the Russian context. There is an urgent need for clinical guidelines and institutional programs that identify and support families where parents experience high levels of PB, regardless of whether they also meet the criteria for other mental health disorders, to promote parental well-being, healthier parent–child interactions, and optimal child development.

## Figures and Tables

**Table 1:** Characteristics of the sample (N = 419).

Characteristics		Mean/N	SD/%	Range
Parental characteristics				
Age (years)		33.34	4.29	23–49
Education	primary/secondary	19	4.5%	
	tertiary	400	95.5%	
Family status	married/cohabiting with a partner	399	95.2%	
	single/divorced	20	4.8%	
Gestational age at childbirth		39.36	1.92	27–42
The number of children in a family	1	252	60.1%	
	2	92	22.0%	
	3	49	11.7%	
	4	18	4.3%	
	5	4	1.0%	
	6	2	0.5%	
	7	2	0.5%	
Mode of birth	Vaginal (including medical interventions	300	71.6%	
	Cesarean (planned/emergency)	119	28.4%	
EPDS total score		9.23	6.01	0–29
Had clinically significant PPD symptoms (score ≥ 10)		148	35%	
BDI total score		8.99	7.66	0–39
Had clinically significant present maternal depressive symptoms (score ≥ 20)		43	10.3%	
PBI total score		39.82	33.51	0–138
PBI score above the cut-off point		68	16.2%	
Child characteristics				
Age (years)		2.24	0.63	1.5–4
Child’s complications during pregnancy/childbirth	No	329	78.5%	
	Yes	90	21.5%	
CBCL checklist				
Internalizing problems		48.15	9.78	29–78
Had borderline/clinically significant internalizing problems (score ≥ 65)		23	5.5%	
Externalizing problems		48.92	8.96	28–80
Had borderline/clinically significant externalizing problems (score ≥ 65)		26	6.2%	
CBCL total score		49.35	9.24	28–76
Had borderline/clinically significant total behavioral problems (score ≥ 65)		22	5.3%	
DSM-oriented scales				
Depressive problems		1.28	1.34	0–9
Anxiety problems		3.21	2.25	0–13
Autism spectrum problems		1.53	1.83	0–11
Attention deficit/Hyperactivity problems		4.57	2.52	0–12
Oppositional defiant problems		2.61	2.05	0–11

EPDS—Edinburgh Postnatal Depression Scale; PPD—postpartum depression; BDI—Beck Depression Inventory; PBI—Parental Burnout Inventory; CBCL—Child Behavior Checklist; DSM—Diagnostic and Statistical Manual of Mental Disorders.

**Table 2:** Multiple linear regression models: association between PB scores at Stage 2 and CBCL scores (Stage 2), excluding and including PPD (Stage 1) and present maternal depression (Stage 2).

Regression Models/Outcomes	β (95%CI)	β (95%CI)	Adjusted R^2^
Model 1. Parental Burnout			
Internalizing problems	0.202 (0.109, 0.295) **		0.10
Externalizing problems	0.309 (0.219, 0.400) **		0.15
Total problems	0.294 (0.204, 0.384) **		0.15
DSM Depressive problems	0.211 (0.116, 0.305) **		0.07
DSM Anxiety problems	0.667 (0.457, 0.877) **		0.10
DSM Autism spectrum problems	0.147 (0.051, 0.242) **		0.05
DSM Attention deficit/hyperactivity problems	0.264 (0.172, 0.356) **		0.12
DSM Oppositional defiant problems	0.208 (0.117, 0.300) **		0.13
Model 2. Parental Burnout (PB) and Postpartum Depression (PPD)			
	PB	PPD	
Internalizing problems	0.181 (0.083, 0.279) **	0.067 (−0.033, 0.168)	0.10
Externalizing problems	0.301 (0.206, 0.397) **	0.025 (−0.073, 0.124)	0.14
Total problems	0.282 (0.187, 0.377) **	0.038 (−0.059, 0.136)	0.15
DSM Depressive problems	0.201 (0.101, 0.301) **	0.031 (−0.071, 0.134)	0.07
DSM Anxiety problems	0.619 (0.397, 0.840) **	0.153 (−0.074, 0.380)	0.10
DSM Autism spectrum problems	0.166 (0.065, 0.266) **	−0.060 (−0.163, 0.044)	0.05
DSM Attention deficit/hyperactivity problems	0.263 (0.165, 0.360) **	0.004 (−0.096, 0.104)	0.11
DSM Oppositional defiant problems	0.213 (0.116, 0.309) **	−0.015 (−0.114, 0.085)	0.13
Model 3. Parental Burnout (PB) and Present Maternal Depression			
	PB	Present Maternal Depression	
Internalizing problems	0.142 (0.030, 0.254) *	0.109 (−0.005, 0.223)	0.10
Externalizing problems	0.292 (0.183, 0.402) **	0.031 (−0.080, 0.143)	0.14
Total problems	0.243 (0.135, 0.352) **	0.092 (−0.019, 0.202)	0.16
DSM Depressive problems	0.150 (0.036, 0.264) **	0.110 (−0.006, 0.225)	0.08
DSM Anxiety problems	0.665 (0.411, 0.919) **	0.003 (−0.255, 0.262)	0.09
DSM Autism spectrum problems	0.149 (0.034, 0.265) *	-0.004 (−0.122, 0.113)	0.05
DSM Attention deficit/hyperactivity problems	0.283 (0.171, 0.394) **	−0.034 (−0.147, 0.079)	0.11
DSM Oppositional defiant problems	0.246 (0.135, 0.356) **	−0.068 (−0.18, 0.045)	0.13

Note. β—standardized regression coefficients from a multiple linear regression model; 95% CI—95% Confidence Interval; PB—parental burnout; PPD—postpartum depression. DSM—The Diagnostic and Statistical Manual of Mental Disorders. All models are adjusted for maternal age at testing, level of education, family status, socioeconomic status, the number of children in a family, mode of birth, gestational age at childbirth, child’s complications during pregnancy/childbirth, and child age at Stage 2. * *p* < 0.05, ** *p* < 0.001.

**Table 3:** Binary logistic regression models: association between PB scores at Stage 2 and child borderline/clinically significant child behavioral problems (Stage 2), excluding and including PPD (Stage 1) and present maternal depression (Stage 2).

Regression Models/Outcomes	OR (95% CI)	OR (95% CI)
Model 1. Parental Burnout		
Internalizing problems	1.70 (1.03, 2.82) *	
Externalizing problems	1.74 (1.05, 2.87) *	
Total problems	1.44 (0.87, 2.37)	
Model 2. Parental Burnout (PB) and Postpartum Depression (PPD)		
	PB	PPD
Internalizing problems	1.54 (0.92, 2.61)	1.36 (0.80, 2.34)
Externalizing problems	1.59 (0.94, 2.67)	1.32 (0.79, 2.23)
Total problems	1.32 (0.79, 2.21)	1.37 (0.79, 2.37)
Model 3. Parental Burnout (PB) and Present Maternal Depression		
	PB	Present Maternal Depression
Internalizing problems	1.42 (0.78, 2.56)	1.36 (0.77, 2.39)
Externalizing problems	1.69 (0.92, 3.14)	1.04 (0.60, 1.79)
Total problems	1.21 (0.67, 2.20)	1.33 (0.75, 2.38)

Note. OR—odds ratio coefficient from the binary logistic regression model; 95% CI—95% Confidence Interval; PB—parental burnout; PPD—postpartum depression. All models are adjusted for maternal age at testing, level of education, family status, socioeconomic status, the number of children in a family, mode of birth, gestational age at childbirth, child’s complications during pregnancy/childbirth, and child age at Stage 2. * *p* < 0.05

## Data Availability

We are ready to provide an anonymized dataset, syntaxes and the survey form (in Russian) by request.

## Supplementary Materials

The following supporting information can be downloaded at https://www.mdpi.com/article/10.3390/children13020176/s1, Table S1: Spearman correlations between parental burnout, postpartum depressive symptoms, present maternal depressive symptoms, and Child Behavior Checklist scales.
