# Supplementary material for: Parental Burnout and Early-Childhood Behavioral Problems: Longitudinal Associations Beyond Maternal Depression
Source: Children (Basel). 2026 Jan 27;13(2):176. doi: 10.3390/children13020176 (PMC12939819; doi:10.3390/children13020176)
Supplement: Supplementary file 1 [file children-13-00176-s001.zip › children-4072355-supplementary.pdf]

Table S1. Spearman correlations between parental burnout, postpartum depressive symptoms, present maternal depressive symptoms, and Child Behavior Checklist scales

	PBI total score	EPDS total score	BDI total score	Internalizing problems	Externalizing problems	Total problems	Depressive problems	Anxiety problems	Autism spectrum problems	Attention deficit/Hyperactivity problems	Oppositional problems
PBI total score		rho=0.36**	rho=0.65**	rho=0.22**	rho=0.32**	rho=0.30**	rho=0.23**	rho=0.30**	rho=0.18**	rho=0.26**	rho=0.28**
EPDS total score	rho=0.36**		rho=0.41**	rho=0.17**	rho=0.17**	rho=0.18**	rho=0.14**	rho=0.15**	rho=0.08	rho=0.13**	rho=0.13**
BDI total score	rho=0.65**	rho=0.41**		rho=0.23**	rho=0.23**	rho=0.26**	rho=0.16**	rho=0.17**	rho=0.11*	rho=0.15**	rho=0.14**
Internalizing problems	rho=0.22**	rho=0.17**	rho=0.23**		rho=0.63**	rho=0.84**	rho=0.46**	rho=0.67**	rho=0.65**	rho=0.47**	rho=0.51**
Externalizing problems	rho=0.32**	rho=0.17**	rho=0.23**	rho=0.63**		rho=0.89**	rho=0.50**	rho=0.46**	rho=0.38**	rho=0.77**	rho=0.71**
Total problems	rho=0.30**	rho=0.18**	rho=0.26**	rho=0.84**	rho=0.89**		rho=0.58**	rho=0.65**	rho=0.53**	rho=0.71**	rho=0.66**
Depressive problems	rho=0.23**	rho=0.14**	rho=0.16**	rho=0.46**	rho=0.50**	rho=0.58**		rho=0.52**	rho=0.40**	rho=0.42**	rho=0.51**
Anxiety problems	rho=0.30**	rho=0.15**	rho=0.17**	rho=0.67**	rho=0.46**	rho=0.65**	rho=0.52**		rho=0.42**	rho=0.40**	rho=0.46**

Autism spectrum problems	rho=0.18**	rho=0.08	rho=0.11*	rho=0.65**	rho=0.38**	rho=0.53**	rho=0.40**	rho=0.42**		rho=0.31**	rho=0.42**
Attention deficit/Hyperactivity problems	rho=0.26**	rho=0.13**	rho=0.15**	rho=0.47**	rho=0.77**	rho=0.71**	rho=0.42**	rho=0.40**	rho=0.31**		rho=0.48**
Oppositional problems	rho=0.28**	rho=0.13**	rho=0.14**	rho=0.51**	rho=0.71**	rho=0.66**	rho=0.51**	rho=0.46**	rho=0.42**	rho=0.48**	

Note. PBI total score = Parental Burnout Inventory total score; EPDS total score = Edinburgh Postnatal Depression Scale total score; BDI total score = Beck Depression Inventory -II total score; Internalizing problems = Internalizing scales of the Child Behavior Checklist 1½-5 score; Externalizing problems = Externalizing scales of the Child Behavior Checklist 1½-5 score; Total problems = sum of all the behavioral problems scales of the Child Behavior Checklist 1½-5; Depressive problems = DSM-oriented depressive problems scale of the Child Behavior Checklist 1½-5; Anxiety problems = DSM-oriented anxiety problems scale of the Child Behavior Checklist 1½-5; Autism spectrum problems = DSM-oriented autism spectrum problems scale of the Child Behavior Checklist 1½-5; Attention deficit/Hyperactivity problems = DSM-oriented attention deficit/hyperactivity problems scale of the Child Behavior Checklist 1½-5; Oppositional problems = DSM-oriented oppositional spectrum problems scale of the Child Behavior Checklist 1½-5.

*p<0.05, **p<0.01
